# Quantitative proteomic dataset of mouse caput epididymal epithelial cells exposed to acrylamide *in vivo*

**DOI:** 10.1016/j.dib.2022.108032

**Published:** 2022-03-08

**Authors:** Natalie A. Trigg, David A. Skerrett-Byrne, Jacinta H. Martin, Geoffry N. De Iuliis, Matthew D. Dun, Shaun D. Roman, Andrew L. Eamens, Brett Nixon

**Affiliations:** a Priority Research Centre for Reproductive Science, School of Environmental and Life Sciences, The University of Newcastle, Callaghan, NSW 2308, Australia; b Hunter Medical Research Institute, New Lambton Heights, NSW 2305, Australia; c Priority Research Centre for Drug Development, School of Environmental and Life Sciences, University of Newcastle, Callaghan, NSW 2308, Australia; d Cancer Signalling Research Group, School of Biomedical Sciences and Pharmacy, Faculty of Health and Medicine, University of Newcastle, Callaghan, NSW 2308, Australia; e Priority Research Centre for Cancer Research Innovation and Translation, Hunter Medical Research Institute, Lambton, NSW 2305, Australia; f School of Chemistry and Molecular Biosciences, The University of Queensland, Brisbane, QLD 4072, Australia

**Keywords:** Acrylamide, Epididymis, Epithelial cells, Proteome, Sperm maturation

## Abstract

This article reports the proteomic legacy of *in vivo* exposure to the xenobiotic, acrylamide, on the epithelial cell population of the proximal segments of the mouse epididymis. Specifically, adult male mice were administered acrylamide (25 mg/kg bw/day) or vehicle control for five consecutive days before dissection of the epididymis. Epididymal epithelial cells were isolated from the proximal (caput) epididymal segment and subjected to quantitative proteomic analysis using multiplexed tandem mass tag (TMT) labeling coupled to mass spectrometry. Here, we report the data generated by this strategy, including the identification of 4405 caput epididymal epithelial cell proteins, approximately 6.8% of which displayed altered expression in response to acrylamide challenge. Our interpretation and discussion of these data features in the article “Acrylamide modulates the mouse epididymal proteome to drive alterations in the sperm small non-coding RNA profile and dysregulate embryo development”

**Specifications table**

**Table:** 

Subject	Omics: Proteomics
Specific subject area	Proteomics, male reproductive tract
Type of data	Figure, RAW data, MassIVE and ProteomeXchange. Supplementary table (Excel) of processed data
How the data were acquired	nanoLC-MS/MS analysis on Q Exactive HF-X Hybrid Quadrupole-Orbitrap coupled to a Dionex Ultimate 3000RSLC nanoflow high-performance liquid chromatography system (Thermo Scientific).
Data format	RAW Analyzed
Description of data collection	Trypsin digested proteins from caput epididymal epithelial cell preparations were labelled with TMT 10plex reagents. The pooled labeled sample was fractionated (11 fractions) prior to LC-MS/MS analysis.
Data source location	The University of Newcastle, Callaghan, Australia
Data accessibility	Mass spectrometry data have been deposited to the ProteomeXchange Consortium via the PRIDE repository (Project identifier: PXD022865). http://dx.doi.org/10.6019/PXD022865
Related research article	Trigg NA, Skerrett-Byrne DA, Xavier MJ, Zhou W, Anderson AL, Stanger SJ, Katen AL, De Iuliis GN, Dun MD, Roman SD, Eamens AL, Nixon B. Acrylamide modulates the mouse epididymal proteome to drive alterations in the sperm small non-coding RNA profile and dysregulate embryo development. Cell Rep. 2021 Oct 5;37(1):109787. doi: 10.1016/j.celrep.2021.109787

**Value of the Data**

• These data provide valuable information on the complexity of the mouse proximal epididymal epithelial cell proteome and the adaptive proteomic response these cells mount following *in vivo* acrylamide exposure.
• These data will benefit researchers investigating the physiological impacts of acrylamide and other compounds (e.g., ethanol or glycidamide) on the male reproductive tract.
• Equally, these data represent a comprehensive catalogue of mouse epididymal epithelial cell proteins, thus forming a highly valuable molecular resource for researchers in examining epididymal function, molecular pathways involved in promoting sperm maturation and/or for the identification of protein biomarkers associated with stressors of the male reproductive tract.
• These data will also be of benefit in the identification of acrylamide responsive proteins in tissues beyond those of the male reproductive tract.

## 1 Data Description

**Fig. 1:**
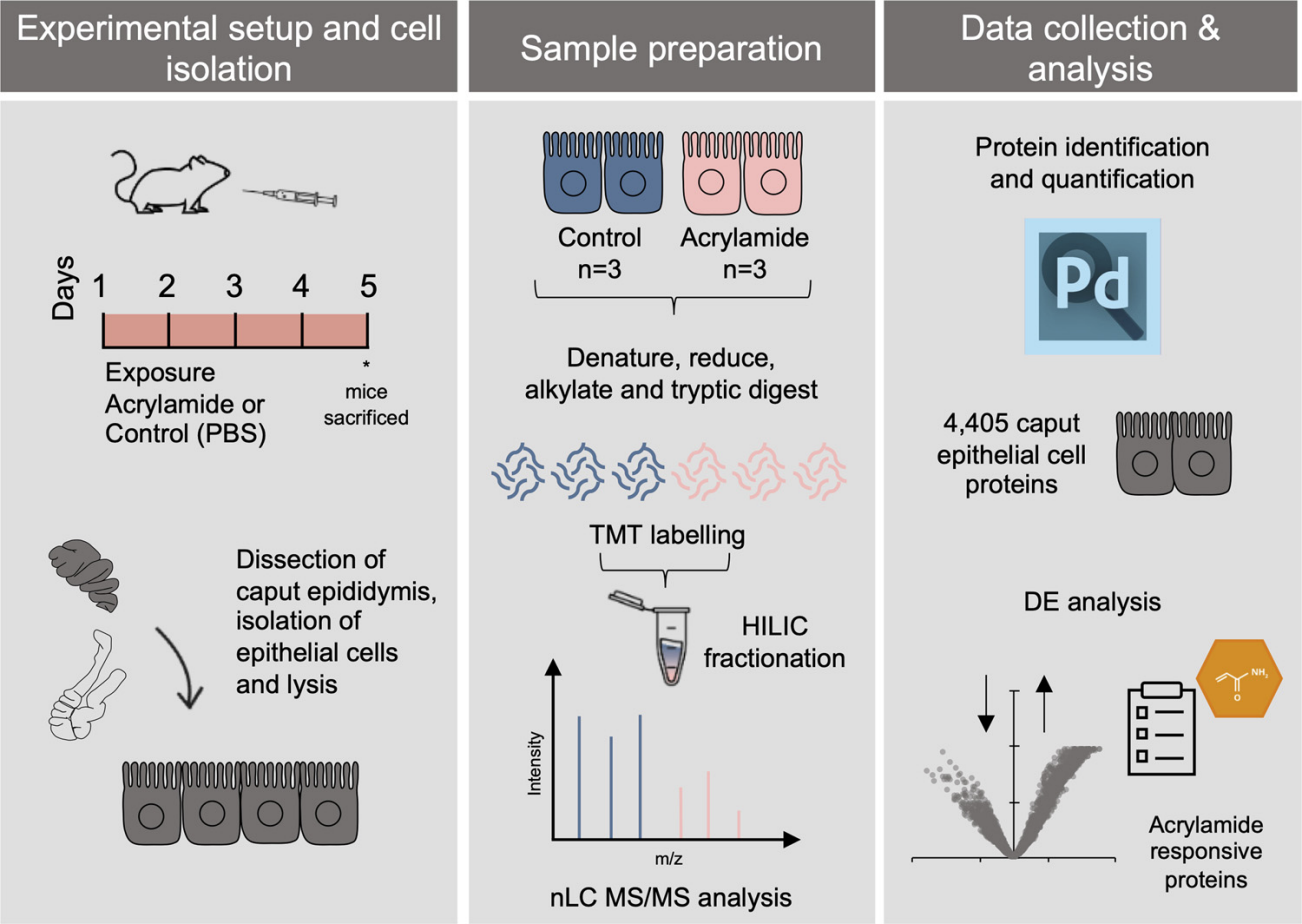
Experimental method. Epididymal epithelial cells were isolated from the proximal epididymis of mice administered either vehicle control or acrylamide (25 mg/kg bw/day). Cells were lysed before proteins were extracted, denatured, reduced, alkylated and digested. Peptides were labeled using isobaric TMT labels and samples were mixed 1:1. Peptides were fractionated into 11 fractions and analyzed on nano-LC MS/MS. Data were processed in Proteome Discoverer 2.4 to identify and quantify proteins. Subsequently, differential expression analysis was performed on the refined protein list.

The files in this article comprise raw and processed data from a comparative proteomics experiment of caput epididymal epithelial cells isolated from male mice exposed to either acrylamide (25 mg/kg bw/day) or vehicle control. The workflow applied for cell isolation, sample preparation and data acquisition and analysis is depicted in Fig. 1 and outlined in the experimental design, materials and methods section below. This proteomic analysis led to the identification of 4405 proteins (Supplementary Table S1), including proteins known to be enriched in the proximal epididymis (DEFB41, SPAG11A/B and LCN8) [1]. Of the total identified epithelial cell proteins, an average of 12.1 peptides (10.8 unique peptides) were identified per protein, with an average peptide coverage of 26.9% per protein. Using a fold-change threshold of ± 1.5 and *p*-value ≤ 0.05 revealed that 6.8% of proteins (302 proteins) displayed altered expression in epididymal epithelial cells from mice exposed to acrylamide. Specifically, the majority of proteins (240 proteins) that displayed differential expression were increased in abundance, equating to 5.4% of all identified proteins, while 1.4% of proteins (62 proteins) were decreased in expression, following acrylamide exposure (Supplementary Table S1). Among the most significantly altered proteins were SERP1, KIF3A and NR3C1, which displayed increased expression following acrylamide exposure and GPX6, RER1 and TSPAN31, which were downregulated in acrylamide exposed epithelial cells. Processed data listing the 4405 proteins identified in caput epididymal epithelial cells and the differential protein expression associated with *in vivo* acrylamide exposure (abundance ratio) are included in this article. Supplementary Table S1 also contains the number of peptides (overall and unique), the percentage of peptide coverage, any detected peptide modifications and the abundance of each identified protein across three biological replicates. These data have been used to explore the proteomic impact of acrylamide exposure on the caput epididymis and interpret the mechanism by which acrylamide exposure leads to an altered microRNA profile of epididymal spermatozoa [2].

## 2 Experimental Design, Materials and Methods

### 2.1 Animals

Adult (8–12 weeks of age) male Swiss mice were obtained from the University of Newcastle's Central Animal House and were housed under a controlled lighting regimen (12 h light, 12 h dark) at 21-22 °C and supplied food *ad libitum*.

### 2.2 Acrylamide exposure regimen

After an acclimatization period of 7 days, male mice received an intraperitoneal injection of acrylamide (25 mg/kg bw/day) or vehicle alone (phosphate buffered saline, PBS) for five consecutive days (100 µl). Mice were euthanized via CO_2_ inhalation on the fifth day, 2-3 h following the final acrylamide injection. Prior to dissection, the mice were perfused with pre-warmed Tris-buffered saline (TBS) to eliminate blood contamination from their vasculature. The caput epididymis was dissected and cleaned of fat and prepared for isolation of epididymal epithelial cells.

### 2.3 Purification of caput epididymal epithelial cells

Dissected caput epididymides were placed in a droplet of Biggers Whitten, and Whittingham (BWW) [3] media composed of 91.5 mM NaCl, 4.6 mM KCl, 1.7 mM CaCl_2_2H_2_O, 1.2 mM KH_2_PO_4_, 1.2 mM MgSO_4_7H_2_O, 25 mM NaHCO_3_, 5.6 mM D-glucose, 0.27 mM sodium pyruvate, 44 mM sodium lactate, 5 U/mL penicillin, 5 μg/mL streptomycin, 20 mM HEPES buffer, and 3.0 mg/mL bovine serum albumin [BSA] (pH 7.4; osmolarity 300 mOsm/kg) and multiple incisions were made in the tissue with a razor blade to allow spermatozoa harbored in the epididymal lumen to disperse. The epididymal tissue was then washed to remove all spermatozoa by subjecting it to agitation and washing three times with sterile Tris buffered saline (TBS). Tissue was subsequently digested with 100 µg/mL trypsin in TBS at 37 °C for 30 min with vigorous shaking (1000 rpm). Clumped tissue sections were collected by centrifugation (800 × g for 5 min) and resuspended in collagenase type II (1.0 mg/mL) in TBS. After incubation for 30 min with shaking at 37 °C the resulting suspension was pipetted up and down to further homogenize it and ensure no observable tissue pieces remained. Once cell disaggregation was completed, the suspension was pelleted via centrifugation and resuspended in Dulbecco's Modified Eagle Medium (DMEM) culture medium containing sodium pyruvate (1 mM), 10% fetal bovine serum, 100 IU/mL penicillin, and 100 µg/mL streptomycin. The cell suspension was incubated in a 6-well plate at 32 °C for 4 h to allow nonepithelial cells to attach to the plate, leaving epididymal epithelial cell aggregates in suspension. Hence, following incubation the supernatant was collected, filtered through a 70 µM membrane, washed, and resuspended in lysis buffer (100 μL of ice-cold 0.1 M Na_2_CO_3_; pH 11.3) supplemented with protease and phosphatase inhibitors (Complete EDTA free; Roche Holding SG, Basel, Switzerland) in preparation for proteomic processing. A single biological replicate was generated by pooling epithelial cells from five to six animals and three such replicates were analyzed. An aliquot of each epithelial cell suspension was assessed by immunocytochemistry using a nuclear stain 4′,6-diamidino-2-phenylindole (DAPI) to ensure that each sample was free of spermatozoa contamination.

### 2.4 Protein digestion and labeling for comparative and quantitative proteomic analysis

Epididymal epithelial cell pellets were prepared for proteomic analysis as previously described [4]. Briefly, thawed cell suspensions were sonicated at 4 °C for 3 × 10 s intervals (100% output power) and subsequently incubated at 4 °C for 1 h. The protein concentration of each sample was determined using a bicinchoninic acid assay (Thermo Fisher Scientific) prior to dilution in a urea solution (6 M urea, 2 M thiourea). Before digestion, each protein sample was reduced and alkylated using 10 mM dithiothreitol (30 min, room temperature) and 20 mM iodoacetamide (30 min, room temperature, in the dark), respectively. Peptide populations were digested with 1:30 Lys-C/Trypsin mix for 3 h at room temperature. The urea concentration was then reduced to below 1 M by addition of 50 mM tetrathylammonium bromide (TEAB; pH 7.8) and digested overnight at 37 °C. Lipids were precipitated using formic acid (2% v/v final concentration) and the resulting peptides were purified using desalting columns (Oasis PRIME HLB; Waters, Rydalmere, NSW, Australia). After digestion and desalting, 100 µg of peptides were labeled using tandem mass tags (TMT) reagents according to the manufacturers protocol (TMT 10plex labels; control 1 = 126, control 2 = 127N, control 3 = 127 C, acrylamide 1 = 129N, acrylamide 2 = 129 C, acrylamide 3 = 130N; Thermo Fisher Scientific).

### 2.5 Tandem mass spectrometry (nanoLC-MS/MS) comparative and quantitative analysis

The TMT-labeled peptide mixture was subjected to fractionation via hydrophilic interaction chromatography (HILIC, 11 fractions) and reverse phase nLC-MS/MS was performed on control and acrylamide treated samples using Q-Exactive HF-X Hybrid Quadrupole-Orbitrap MS coupled to a Dionex Ultimate 3000RSLC nanflow high performance liquid chromatography system (Thermo Fisher Scientific). Peptides were separated using an in-house packed column, SGE MyCapLC Kit 300 µm × 150 mm, employing a gradient of acetonitrile (300 nL/min; 3–25%, 55 min; 25–60%, 70 min; 60–98%, 15 min). A Q-Exactive HF-X-MS System was operated in full scan mode (300–1650 m/z, resolationg 60,000 FWHM), at an automatic gain control target of 3 × 10^6^ (max fill time 50 ms), with 15 data dependant acquisitions (MS/MS) selected for higher energy collision dissociation fragmentation (automatic gain control target 1 × 10^6^, max fill time 120 ms, normalized collisional energy of 32, and fragment resolution 45,000 FWHM.

### 2.6 Proteomic data processing

The database for *Mus Musculus* downloaded from UniProt (25,260 sequences, downloaded 12th November 2019 including reviewed entries and their canonical and isoform sequences) was searched against the raw files using Proteome Discoverer (PD) software (version 2.4, Thermo Fisher Scientific) and the SEQUEST HT search algorithm. Searches were performed using the following parameters: two maximum missed cleavages for trypsin, a mass tolerance of precursor mass and fragment mass of 10 ppm and 0.02 Da, respectively and trypsin was specified as the cleavage enzyme. To evaluate the false discovery rate of peptide identification, the corresponding reversed database was interrogated using Percolator. For this, the target-decoy search approach was used to obtain q-values. PD Normalization node was utilized prior to statistical testing, whereby it sums the peptide group abundances for each TMT label and determines the maximum sum for all labels. The normalization factor is the factor of the sum of the sample and the maximum sum in all labels [4,5]. Fold changes between control and acrylamide sample groups were determined by PD, whereby the program calculates the protein ratios (abundance ratio) as the geometric median of all peptide group ratios. Statistical analyses were completed using a Student's *t* test, with *p* ≤ 0.05 considered significant. The protein lists were exported from PD as Excel files and the final list of proteins was refined to include proteins with a quantitative value in all three biological replicates and a minimum of 2 unique peptides.

## Ethics Statements

All experiments were conducted with the approval of The University of Newcastle's Animal Care and Ethics Committee (ACEC, approval number A-2017-726).

## CRediT authorship contribution statement

**Natalie A. Trigg:** Conceptualization, Methodology, Investigation, Formal analysis, Writing – original draft, Writing – review & editing. **David A. Skerrett-Byrne:** Investigation, Formal analysis, Writing – review & editing. **Jacinta H. Martin:** Writing – review & editing. **Geoffry N. De Iuliis:** Conceptualization, Supervision, Methodology, Writing – review & editing. **Matthew D. Dun:** Methodology, Funding acquisition, Conceptualization, Writing – review & editing. **Shaun D. Roman:** Conceptualization, Methodology, Supervision, Writing – review & editing. **Andrew L. Eamens:** Conceptualization, Supervision, Methodology, Writing – review & editing. **Brett Nixon:** Conceptualization, Methodology, Writing – original draft, Writing – review & editing, Supervision, Funding acquisition.

## Declaration of Competing Interest

The authors declare that they have no known competing financial interests or personal relationships that could have appeared to influence the work reported in this paper.

## Appendix Supplementary materials

Image, application 1

Image, application 2
